# Supplementary figures and images for: A Vimentin-Targeting Oral Compound with Host-Directed Antiviral and Anti-Inflammatory Actions Addresses Multiple Features of COVID-19 and Related Diseases
Source: mBio. 2021 Oct 12;12(5):e02542-21. doi: 10.1128/mBio.02542-21 (PMC8510534; doi:10.1128/mBio.02542-21)

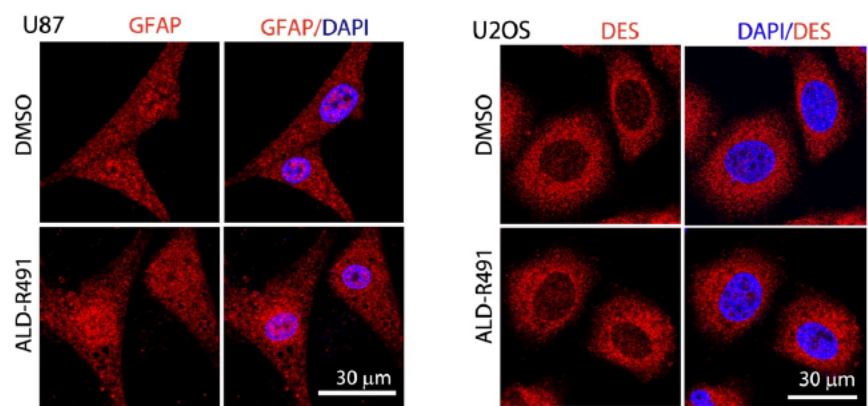

**Fig. S1.**

Supplement: FIG S1 [file mbio.02542-21-sf001.pdf]

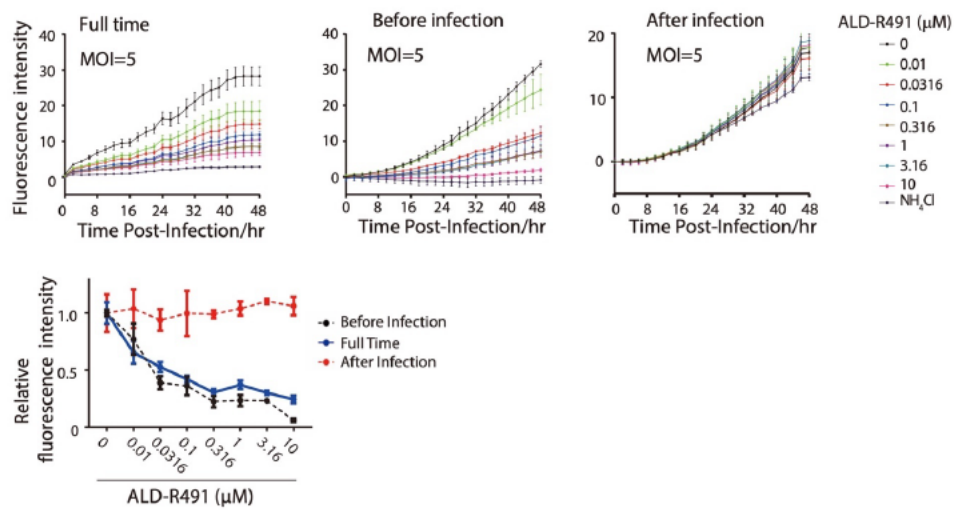

**Fig. S2.**

Supplement: FIG S2 [file mbio.02542-21-sf002.pdf]

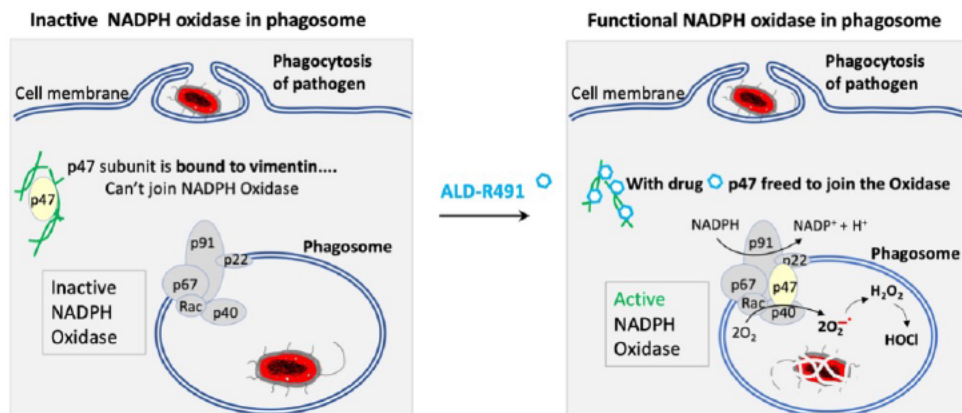

9

10 **Fig. S3.**

11

Supplement: FIG S3 [file mbio.02542-21-sf003.pdf]

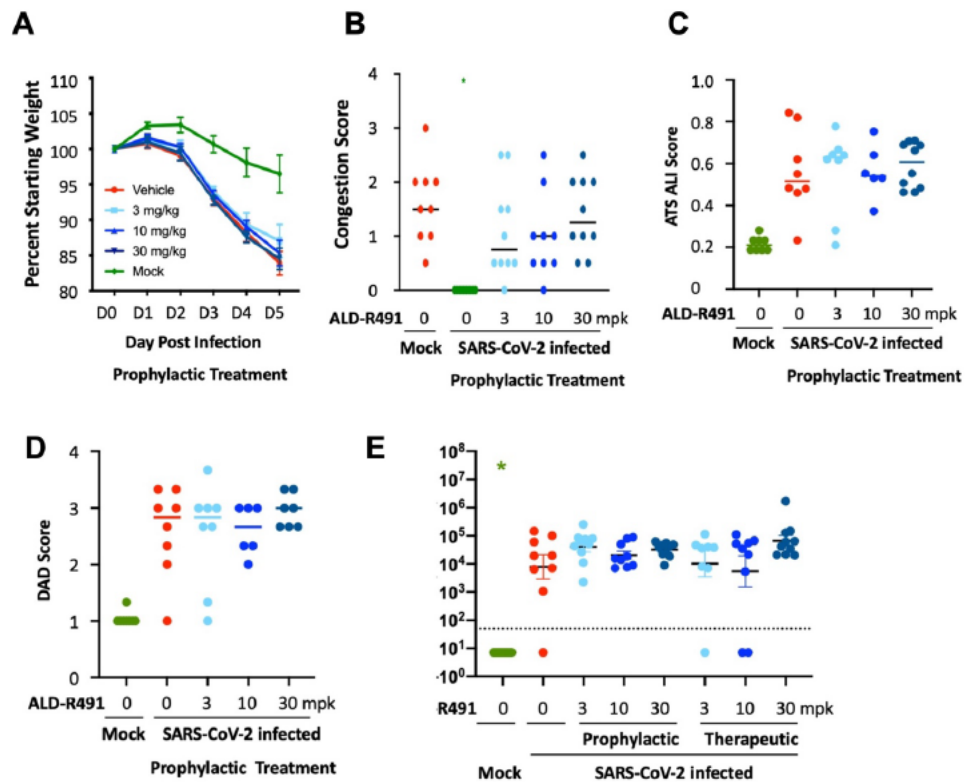

Fig. S5.

Supplement: FIG S5 [file mbio.02542-21-sf005.pdf]

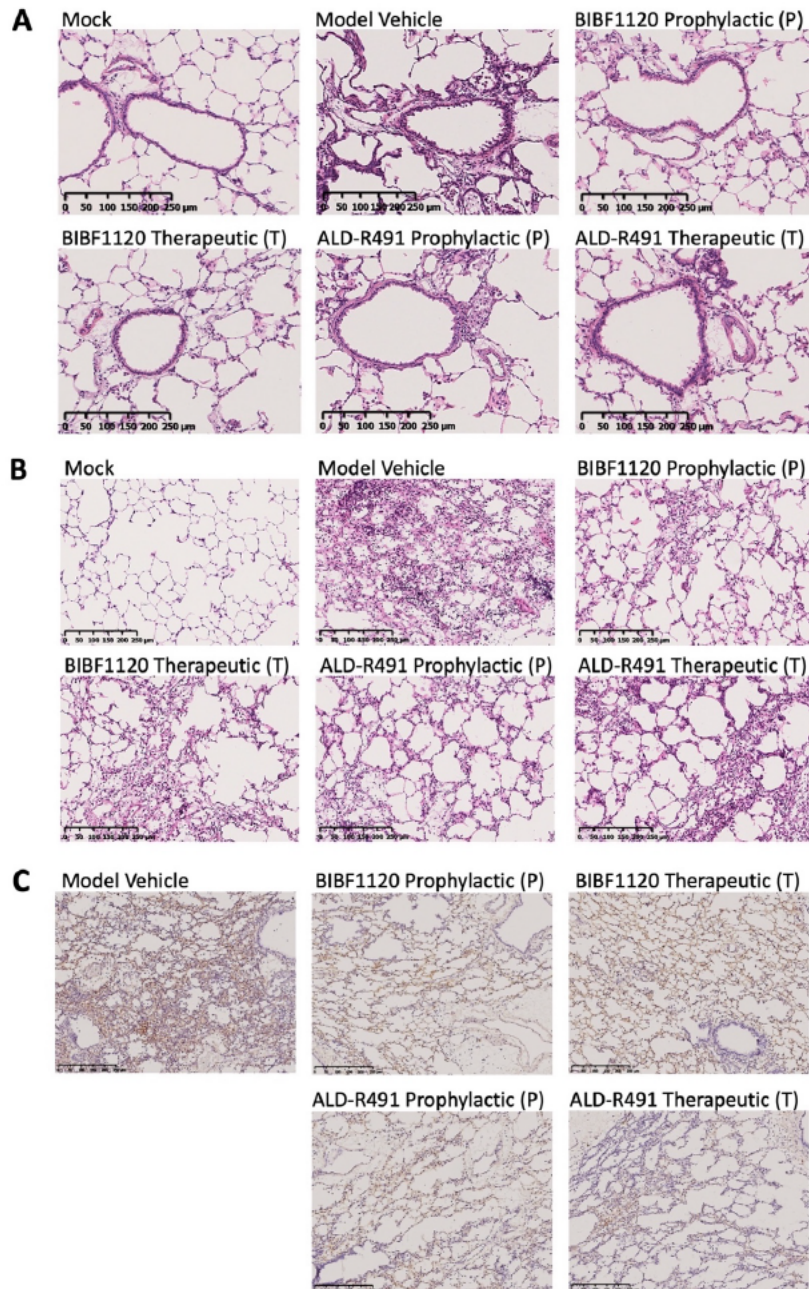

20

21 **Fig. S6.**

Supplement: FIG S6 [file mbio.02542-21-sf006.pdf]
