# Supplementary material for: A Vimentin-Targeting Oral Compound with Host-Directed Antiviral and Anti-Inflammatory Actions Addresses Multiple Features of COVID-19 and Related Diseases
Source: mBio. 2021 Oct 12;12(5):e02542-21. doi: 10.1128/mBio.02542-21 (PMC8510534; doi:10.1128/mBio.02542-21)
Supplement: FIG S4 [file mbio.02542-21-sf004.pdf]

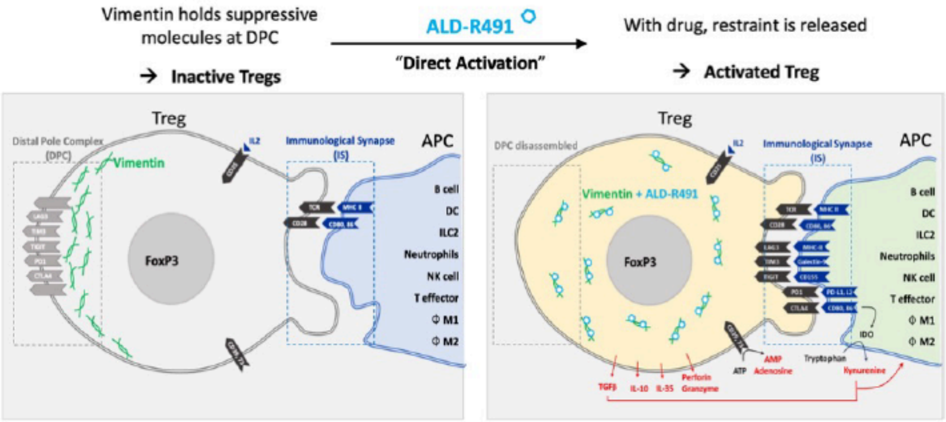
Vimentin holds suppressive molecules at DPC
ALD-R491
"Direct Activation"
With drug, restraint is released
→ Inactive Tregs
→ Activated Treg
Treg
Distal Pole Complex (DPC)
Vimentin
IL2
Immunological Synapse (IS)
APC
FoxP3
B cell
DC
ILC2
Neutrophils
NK cell
T effector
Φ M1
Φ M2
Treg
DPC disassembled
Vimentin + ALD-R491
Immunological Synapse (IS)
APC
FoxP3
B cell
DC
ILC2
Neutrophils
NK cell
T effector
Φ M1
Φ M2
IDO
ATP
AMP
Adenosine
Tryptophan
Kynurenine
TGFβ
IL-10
IL-35
Perforin
Granzyme

Fig. S4.
